# Efficient Lignin Precipitation from Softwood Black Liquor Using Organic Acids for Sustainable Valorization

**DOI:** 10.3390/polym17070926

**Published:** 2025-03-29

**Authors:** Elsa Duret, Luanna C. R. de Moura, Amaia Morales, Jalel Labidi, Eduardo Robles, Fatima Charrier-El Bouhtoury

**Affiliations:** 1 CNRS, University of Pau and Adour Countries, IPREM-UMR 5254, 40004 Mont de Marsan, France; elsa.duret@univ-pau.fr (E.D.);; 2 Chemical and Environmental Engineering Department, Faculty of Engineering Gipuzkoa, University of the Basque Country UPV/EHU, Plaza Europa 1, 20018 Donostia, Spain; amaia.morales@ehu.eus (A.M.); jalel.labidi@ehu.eus (J.L.)

**Keywords:** lignin, precipitation, organic acids, black liquor, softwood

## Abstract

The chemical industry’s transition towards sustainability necessitates the development of eco-friendly processes that can replace petrochemical derivatives. Lignin, the second most abundant plant polymer, has potential as a renewable alternative to phenolic compounds. This study investigates lignin precipitation from softwood black liquor using five organic acids (acetic, citric, lactic, malic, and oxalic) as a sustainable alternative to sulfuric acid. The precipitated lignins were subjected to comprehensive chemical and thermal characterization, revealing higher total phenolic content and enhanced reactivity when organic acids were employed. Notably, organic acid-precipitated lignins demonstrated comparable or superior purity, with ash contents below 0.50%, compared to 3.28% observed for sulfuric acid-precipitated lignin. These findings suggest that organic acids are a viable and greener alternative for lignin precipitation, promoting higher purity and yield, thus supporting lignin valorization efforts.

## 1. Introduction

In the context of green chemistry and sustainable development, the demand for environmentally friendly chemical products and processes is growing [1]. In this regard, lignin emerges as a promising candidate for biobased materials and renewable chemicals due to its abundance in biomass, second only to cellulose. Lignin is part of the plant tissue, working for several functions, including its role as the glue that maintains cellulose and hemicellulose together [2]. Lignin presents a complex structure, and its molecule possesses a variety of active groups, such as aromatic, phenolic hydroxyl, and alcoholic hydroxyl groups [3]. Nevertheless, lignin is mainly composed of phenylpropane units, especially p-coumaryl (p-hydroxyphenyl, H unit), coniferyl (guaiacyl, G unit), and sinapyl (syringyl, S unit) alcohols randomly arranged via non-hydrolyzable linkages [4,5]. The chemical structure of lignin exhibits diversity according to variations in plant species. In softwoods, G units predominate, while hardwoods are richer in S units [3]. The extraction process is another factor influencing lignin composition, structure, molecular weight, and physical properties [6,7].

Lignin’s complex and heterogeneous structure has historically hindered its research and application compared to cellulose and hemicellulose [8]. However, its potential for biofuel production and as a renewable source of phenolic compounds, traditionally derived from petrochemicals, has driven advancements in lignin research [9,10,11]. Lignin must exhibit greater predictability and reduced molecular weight to enhance its usability. Various strategies have been explored to achieve this, including fractionation, which isolates molecular weight fractions with defined properties. Traditional fractionation methods such as Sulfite, Kraft, and Soda pulping [12], alongside newer approaches such as selective precipitation, ultrafiltration, and microbial degradation and transformation, facilitate lignin extraction from black liquor, unlocking applications in biofuels and high-value products [10,13,14]. Depolymerization is considered the most promising route, as it converts lignin into low-molecular-weight compounds, including vanillin, hydroxylated aromatics, aldehydes, and aliphatic acids. This process is categorized into five main strategies: reductive, oxidative, acid- or base-catalyzed, solvolytic, and thermal depolymerization [7,15,16,17,18]. Lignin valorization could become a valuable tool for chemical sustainable development due to its properties, such as thermal stability, high biodegradability, and carbon-rich composition [19].

Despite its remarkable properties, lignin remains an underutilized product in industrial applications, with most of it burned for energy in biorefineries and the pulp and paper industry [20]. The pulp and paper industry’s main objective is removing lignin and other non-cellulosic components to obtain high-quality pulp, a process predominantly carried out through the Kraft method [19]. Due to its elevated calorific value, this process generates black liquor, a lignin-rich by-product traditionally used as an internal energy source [2,21]. Every year, over 50 million tons of lignin are removed from biomass, but only 5% is used for applications other than energy [22]. Moreover, it has been estimated that lignin production will increase to 225 million tons per year by 2030 due to biofuel production [5], underscoring the need for alternative value-added applications.

Lignin can be extracted from black liquor by ultrafiltration or precipitation [23]. The precipitation occurs by reducing the pH of the black liquor using sulfuric acid or CO_2_. The use of CO_2_ is mainly present in the recently developed LignoBoost^®^ process, which provides a superior lignin quality. This process is carried out in two stages, first lowering the pH to 8–9 with CO_2_, followed by a second reduction to pH 2–4 with sulfuric acid [24]. However, the use of sulfuric acid may be challenging nowadays. In addition to being dangerous, using this acid during precipitation releases toxic gases such as SO_2_ and H_2_S [25]. These drawbacks, added to the perspectives of production growth for the following decades, promote increasing interest in the search and development of greener recovery routes for lignin. Recent studies have proposed organic acids as more sustainable alternatives for lignin precipitation routes while being less costly and introducing fewer impurities in the resulting lignin [25]. Da Silva and collaborators [26] precipitated lignin from industrial hardwood black liquor with acetic, citric, and lactic acids and made a thorough characterization. The authors found that the obtained lignins presented higher purity than those obtained with sulfuric acid. They also found structural features that contribute to higher antioxidant activity than commercial synthetic antioxidants. Another study precipitated lignin with acetic, citric, and oxalic acid, obtaining lignin of higher purity with citric acid but with a lower yield [27]. These studies provided important insights into greener routes for lignin precipitation and opened many possibilities worth exploring.

Nevertheless, more light is needed on the mechanisms of precipitation as the comparability of these methods remains complicated, as lignins precipitate at different pHs depending on the acid used, making the comparability between them difficult. Several factors contribute to this limitation, with the complexity of lignin’s structure and the variability of pulping processes being among the most significant. Therefore, investigating the precipitation behavior of lignin from black liquor derived from different regions, feedstocks, and processing conditions, alongside a comprehensive characterization of lignin obtained through various organic acids at different pH levels, represents a promising avenue for advancing scientific understanding. The accumulation of such studies may contribute to a more statistically robust understanding of lignin recovery dynamics, ultimately providing more reliable insights into the feasibility of replacing inorganic acids in lignin recovery processes on a larger scale.

In this context, the present study aims to contribute to this growing body of knowledge by focusing on the characterization of softwood black liquor, followed by the precipitation of lignin using different organic acids, namely acetic, citric, lactic, malic, and oxalic at a fixed pH. The resulting lignins were subjected to comprehensive chemical and physical characterizations. The ultimate goal is to contribute evidence showing that organic acids could facilitate lignin production with higher purity, increased yield, and favorable properties. In addition, using organic acids to replace sulfuric acid would enable the development of a more environmentally friendly process.

## 2. Materials and Methods

### 2.1. Chemicals Employed

The industrial softwood black liquor (BL) used in the present study was gracefully donated by a local company (France) and was obtained by the Kraft process on maritime pine (Pinus pinaster). Citric acid 99% (CAS No. 77-92-9), malic acid 99% (CAS No. 97-67-6), oxalic acid 99% (CAS No. 144 62 7), sulfuric acid 96% (CAS No. 7664-93-9), butylated hydroxytoluene (BHT) 99% (CAS No. 128-37 0), and Trolox (CAS No. 53188-07-1) were purchased from Thermo Fisher Scientific (Illkirch-Graffenstaden, France). Lactic acid (CAS No. 50-21-5) and sodium carbonate (CAS No. 497-19-8) were obtained from Honeywell. Hydrochloric acid 37% (CAS No. 7647-01-0) and gallic acid (CAS No. 149-91-7) were products of Acros Organics(Illkirch-Graffenstaden, France). The Folin–Ciocalteu reagent was bought from Merck. Methanol (CAS No. 67-56-1), acetic acid glacial (CAS No. 64-19-7), and N, N-Diméthylformamide (DMF) (CAS No. 68-12-2) were purchased from Fisher Scientific. Dimethyl sulfoxide (DMSO) (CAS No. 67-68-5), chloroform-d (CAS No. 865-49-6), pyridine (CAS No. 110-86-1), Chromium (III) acetylacetonate (Cr(acac)3) (CAS No. 21679-31-2), N-hydroxy-5-norbornene-2,3-dicarboxylic acid imide (NHND) (CAS No. 21715-90-2), 2-Chloro-4,4,5,5-tetramethyl-1,3,2-dioxaphospholane (TMDP) (CAS No. 14812-59-0), and DMSO-d6 (CAS No. 2206-27-1) were obtained from Sigma Aldrich Chimie (St. Quentin Fallavier, France). Lithium bromide (LiBr) (CAS No. 7550-35-8) was purchased from Panreac (Barcelona, Spain), and 2,2-diphenyl-1-picrylhydrazyl (DPPH) (CAS No. 1898-66-4) was bought from Alfa Aesar (Schiltigheim, France).

### 2.2. Black Liquor Characterization

Density was determined by measuring the weight of a known volume of black liquor, and the pH was measured using a pH meter (Antylia Scientific Vernon Hills, Chicago, IL, USA). Total dry solid (TDS) content was determined by drying 5 ± 0.001 g of the black liquor at 105 ± 3 °C for 24 h. TDS was calculated as the ratio of the weight of the recovered dry solid to the initial liquid fraction. Ash, volatile matter, and fixed carbon content were determined using a calcination furnace (Carbolite Gero, Neuhausen, Germany). The black liquor was oven-dried at 105 ± 3 °C for 24 h to determine moisture content. It was then heated to 575 °C for 10 min with the lid on. After this time and 4 h in the desiccator to cool, the weight was recovered to calculate the percentage of lost volatile substances. The crucibles were returned to the calcination furnace at 575 °C without the lid until no carbon was observed inside the crucibles. After each heating, the crucible containing the powder was cooled in a desiccator to constant weight.

Fourier Transformed Infrared (FTIR) spectra were recorded using a Jasco FT/IR-4700 infrared spectrometer equipped with an ATR (Attenuated Total Reflection, Jasco France, Lisses, France) unit at room temperature. Before analysis, samples were ground into a fine powder using a ceramic mortar. At the equipment, a small amount of powder was placed over the crystal, covering it completely. Measurements were carried out in absorbance mode with 64 scans employing a resolution of 4 cm^−1^ in a range of 4000–400 cm^−1^. Spectral Manager software (Jasco) was used to reduce noise and convert the acquired spectrum into text format for further processing.

The black liquor was also subjected to quantitative acid hydrolysis (QAH) following the NREL TP-510-42618 to determine acid-insoluble lignin (AIL or Klason lignin), acid-soluble lignin (ASL), and carbohydrate content. A first acid hydrolysis of the samples was performed as follows: 300.0 ± 10.0 mg of samples were mixed with 3.00 ± 0.01 mL of 72% H_2_SO4 and placed in a water bath at 30 ± 3 °C for 60 ± 5 min. The samples were then diluted with distilled water to a concentration of 4% H_2_SO_4_ before being autoclaved for 1 h at 121 °C for a second acid hydrolysis. After this time, the solid suspension was filtered using a gooch filter crucible (n°3). The filtrate was recovered to determine the acid-soluble lignin and carbohydrate content. The solid residue was washed several times with distilled water and then dried at 105 °C for 24 h to determine the amount of Klason lignin. ASL was determined by measuring the UV absorbance of the filtrate using a V-730 UV Vis spectrophotometer (Jasco, Tokyo, Japan) at 205 nm. The blank was performed with 4% H_2_SO_4_, and an acid-soluble lignin absorptivity value of 110 L/g cm was used. The carbohydrate content was determined by analyzing the filtrate by High-Performance Liquid Chromatography (HPLC) (LC Net II/ADC chromatograph, Jasco France) equipped with a refractive index detector (RI-2031Plus) and a photodiode array detector (MD-2018Plus). The column used was 300 × 7.8 mm Aminex HPX-87H (Bio-Rad Laboratories, Hercules, CA, USA), with a flow rate of 0.6 mL/min at 50 °C and an injection volume of 20 µL. The mobile phase used was a 0.005 M H_2_SO_4_ solution.

Finally, the thermal properties of black liquor were also studied by thermogravimetric analysis (TGA) using a Q500 thermogravimeter (TA Instrument, Guyancourt, France). Between 5 and 10 mg of dried black liquor was heated in an open platinum pan at 10 °C/min from 30 °C to 800 °C under a 40 mL/min nitrogen flow rate.

### 2.3. Lignin Precipitation Using Organic Acids

Five organic acids were used to precipitate the lignin: acetic, citric, lactic, malic, and oxalic. These acids were selected due to lower cost, favorable environmental profiles, different acid strengths, and correlatable data in the literature [26,27,28,29,30]. For further comparison, sulfuric and hydrochloric acids, which are commonly used in industrial-scale lignin production, were also included. The acids were gradually added to the weighed black liquor sample until they reached a stable pH of 2. This pH value was maintained consistently across all acids to ensure a direct comparison, as the pH typically varies in the literature according to the acid used. This was based on studies suggesting that 80% of lignin precipitation commonly occurs when black liquor is acidified to around pH 2, and this pH provides a fair yield and quality response when using sulfuric or hydrochloric acids [31,32]. A pH of 2 was selected to have a comparable precipitation parameter for the different organic acids. Liquid acids (acetic, lactic, sulfuric, and hydrochloric) were added using a burette, while solid acids (citric, malic, and oxalic) were poured directly into the black liquor using a spatula. The pH meter was kept in the solution throughout the experiment, and the pH decrease was always observed. The total amount of each acid added to achieve a solution of pH 2 was recorded. The solid precipitates were then collected using a 0.45 μm nylon filter membrane, and samples were washed with distilled water until a stable neutral pH was reached. The lignin precipitated was oven-dried at 45 °C until constant weight, which was noted for yield determination. Samples were recovered and stored until characterization. The lignin from acetic, citric, lactic, malic, oxalic, sulfuric, and hydrochloric acids were identified by the following codes: AL, CL, LL, ML, OL, SL, and HL, respectively.

### 2.4. Lignins Characterization

Samples recovered were assessed for yield and then characterized using different techniques to assess the lignin quality obtained by the different acids. Many characterizations followed a similar protocol to item 2.2.

#### 2.4.1. Chemical Characterization

FTIR analysis of precipitated lignins was carried out according to the protocol and parameters described for black liquor characterization (item 2.2).

The total phenolic content of the samples was determined using the Folin–Ciocalteu method [33]. First, 0.2 mL of lignin solution (2 g/L in DMSO) was mixed with 1 mL of Folin–Ciocalteu reagent and 2 mL of sodium carbonate solution (200 g/L in water). Water was added to these reagents until a total volume of 20 mL. The mixture was placed in a water bath at 40 °C for 30 min. The absorbance was then read at 750 nm using a UV-visible spectrophotometer (Secoman, Besançon, France). Gallic acid solutions (100–1000 mg/L) were used as calibration curves. Results are expressed as micrograms of gallic acid equivalent per gram of dry lignin (µg GAE/g dry lignin).

The antioxidant activity of the samples was evaluated using the method described by Brand-Williams and his collaborators [34] with some modifications. Lignins were diluted with DMSO between 0 and 0.04 mg/mL.

The antioxidant activity of the samples was evaluated using the method described by Brand-Williams and his collaborators [17] with some modifications. Lignins were diluted with DMSO between 0 and 0.04 mg/mL. For the reaction, 3 mL of diluted lignin was mixed with 3 mL of DPPH (39.4 mg/L) before being placed in the dark for 30 min. The absorbance was then read at 517 nm using a UV-visible spectrophotometer (Secoman, France). DMSO was used as blank. The following formula (Equation (1)) was used to calculate the inhibition percentage:

I (%) = [(A0 − AS)/A0] × 100(1)

where A0 was the absorbance of the initial dilution (3 mL of DMSO + 3 mL of DPPH solution) and AS was the absorbance of the sample (3 mL of the sample at different concentrations + 3 mL of DPPH solution). These values were used to plot a graph: inhibition percentage versus concentration, where a tendency curve was obtained. The concentration to inhibit 50% of the DPPH radical (IC50) was then calculated as Equation (2), as follows:

IC50 (%) = [(50 − b)/a] × 100(2)

where “a” and “b” are the slope and the interception of that tendency curve, respectively. BHT and Trolox, two commercial antioxidants, were used as comparisons. They were diluted between 0 and 0.1 mg/mL, and the same protocol was used, replacing DMSO with methanol.

Gel permeation chromatography (GPC) was used to determine the weight-average molar mass (Mw), the number-average molecular weights (Mn), and the polydispersity index (Đ) of the lignins using a JASCO LC-Net ll/ADC (Japan) equipped with a refractive index detector (RI-2031Plus) and two Polar-Gel M columns (300 × 7.5 mm). The samples were prepared by dissolving 25 mg of lignin powder in 5 mL of N, N-dimethylformamide (DMF). For the analysis, 20 µL of lignin solution was injected, and DMF with 0.1% lithium bromide (LiBr) was used as the mobile phase with a flow rate of 0.7 mL/min. The oven temperature was set at 40 °C. The calibration was conducted using polystyrene standards.

The purity of the lignin was evaluated by determining the acid-insoluble lignin (AIL or Klason lignin), acid-soluble lignin (ASL), and carbohydrate content. The protocol used was based on the same approach mentioned above for black liquor characterization (item 2.2), with a few modifications. Mixed were 250.0 ± 5.0 mg of lignin and 2.50 ± 0.01 mL of 72% H_2_SO_4_ solution. This solution was then placed in a water bath at 30 ± 3 °C for 60 ± 5 min. During this time, the mixture was stirred every 10 min without removing the sample from the water bath. After this initial acid hydrolysis, the solution was diluted by adding 12% H_2_SO_4_ until the total volume reached 24.2 mL. The resulting solution was transferred to an autoclave and subjected to a one-hour treatment at 121 °C. Following this second acid hydrolysis, vacuum filtration was conducted using a gooch filter crucible (n°3). The filtrate was recorded for further analysis to determine the ASL and carbohydrate content. The solid portion was washed repeatedly with distilled water until a neutral pH was attained and then dried in an oven at 105 ± 3 °C for 24 h. The recovered filtrate was diluted with a 12% H_2_SO_4_ solution before measuring its absorbance, following the same procedure described for black liquor analysis. AIL and carbohydrates were determined using the same protocol employed for black liquor characterization (item 2.2).

^31^P Nuclear magnetic resonance (NMR) spectroscopy determined the hydroxyl groups present in the lignin structure. Prior to analysis, sample preparation was required. First, the solvent was prepared by mixing deuterated chloroform and pyridine in a ratio of 1:1.6 (*v*/*v*). Then, 30 mg of lignin was dissolved in 0.5 mL of solvent and 0.1 mL of internal standard. After complete dissolution of the lignin, 0.1 mL of TMDP was added to phosphorylate the hydroxyl groups. The internal standard was a solution of Cr(acac)3 at 5 mg/L and NHND at 18 mg/mL in the previous solvent (deuterated chloroform and pyridine). Spectra of lignins were reordered using a Bruker AVANCE 500 MHz, fixing the parameters as recommended by Meng and co-workers [35].

Ash content was determined using the same protocol described for black liquor characterization (item 2.2).

#### 2.4.2. Thermal Characterization

The thermal stability of the lignin was estimated using thermogravimetric analysis (TGA). The protocol described in the black liquor characterization (item 2.2) was also employed with the precipitated lignins.

## 3. Results and Discussion

The black liquor characteristics and composition can vary according to the raw material (softwood or hardwood) and the process adopted by the pulp and paper plant [36,37]. Table 1 shows the data obtained from the characterization of this black liquor. The black liquor obtained from the pulping process exhibited a high pH value of 12.77 ± 0.46, typical of Kraft pulping processes known for their highly alkaline conditions. The density of the liquor was measured at 1.14 ± 0.00 g/mL, consistent with the dense, viscous nature of black liquor due to its high solids content. The total dry solids content was 21.89 ± 0.06%, indicating a significant concentration of organic and inorganic materials and within the range expected, as it typically ranges between 15% and 30%. The acid-insoluble lignin (AIL) content of 27.31 ± 0.36% was within the expected range, although slightly higher than some literature values, which may be attributed to differences in the raw material or pulping conditions. The high AIL content is particularly significant, representing the fraction of lignin that can be effectively precipitated. The ability to recover a substantial amount of lignin from the black liquor not only adds value to the pulping process but also contributes to the industry’s sustainability by providing a renewable source of lignin for various applications. The AIL value will be used as a reference to determine the precipitation yield with the selected acids.

### 3.1. Lignin Precipitation

**Figure 1:**
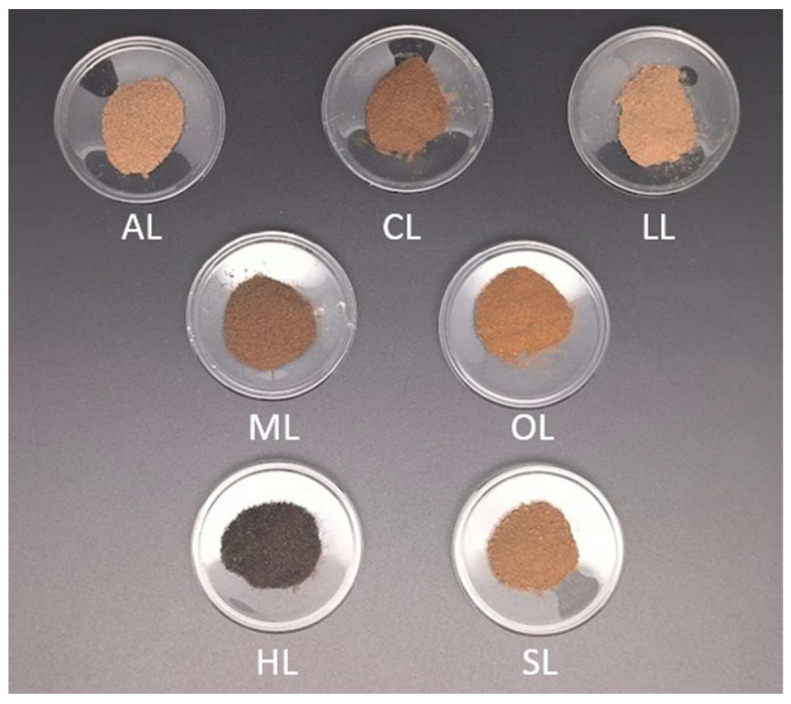
Lignins precipitated with different acids presenting different colors (AL: acetic acid, CL: citric acid, LL: lactic acid, ML: malic acid, OL: oxalic acid, HL: hydrochloric acid, and SL: sulfuric acid).

Depending on the type of acid used for lignin precipitation, it took between one and two hours to decrease the pH to 2 ± 0.2. During this process, it was observed that if the pH was decreased abruptly, there was significant foam formation, while a slight decrease reduced foam volume. Depending on the acid used, the final color of the solution would vary in tonalities of browns (Figure 1). These color differences may be due to a combination of factors during precipitation, as strong or polyprotic acids (as sulfuric, hydrochloric, and oxalic) can induce C-C condensation in phenolic units or slight oxidation giving place to the formation of quinones, which can be more or less dark depending on the degree of conjugation. Other factors are the molecular weight and distribution, the aggregation state due to the oven drying technique, and the eventual oxidation, conjugation, and unsaturation degree of lignin [38]. During the filtration process, differences between acids were also observed. Lignins precipitated with citric and lactic acids took longer to filtrate, while oxalic-precipitated lignin was faster than the others. Da Silva and co-authors noted a similar observation regarding lactic acid in their work [26]. However, other studies [26,39] had a different experience regarding the filterability using citric acid, where an increase in the filtration speed was mentioned when compared to sulfuric acid lignin. The use of low pH in the present study may be the main reason for the differences observed in filterability, as the pH may have resulted in finer particles than the size of the particles in other studies that used greater pH values [40].

**Figure 2:**
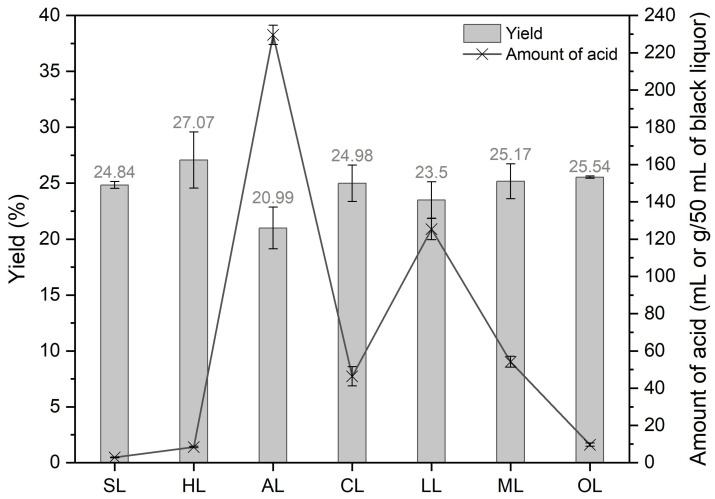
Lignin precipitation yields and amount of acid used.

After neutralization and filtration, it was possible to determine the yield of the precipitated lignin, presented in Figure 2, and the amount of acid used for each precipitated lignin. The yield was calculated by calculating the amount of acid-insoluble lignin (AIL) obtained during the characterization of black liquor. Precipitating lignin with organic acids produces yields similar to those obtained with inorganic acids in the same experimental settings. However, the amount of acid used to lower the pH to 2 is more important for organic acids. Among them, the relation between the amount of acid and yield was more advantageous regarding oxalic acid.

In contrast, acetic acid requires comparatively more acid, while the yield is the lowest. A possible explanation for these differences could be related to the acid strength. Acids with a low pKa are considered strong acids and require less to lower the pH to 2. The acids used are classified from strongest to weakest according to their pKa: hydrochloric, sulfuric, oxalic, citric, malic, lactic, and acetic acid. Except for sulfuric acid and hydrochloric acid (pKa very close), the same order can be observed regarding the quantity of acid used. Other studies [41,42] have shown that the yield obtained depends on the type of acid used: monoprotic, diprotic, or triprotic. These studies indicate that the greater the number of protons an acid can release, the higher the yield. In the present case, this assumption is not fully respected, as citric acid (triprotic) does not provide a better yield than oxalic acid (diprotic) or hydrochloric acid (monoprotic). However, another study [27] used oxalic, acetic, citric, and sulfuric acids to precipitate lignin and showed that citric acid also yielded higher yields. They obtained 10.19 g/100 mL of black liquor, compared with 7.45 g/100 mL in the present study. It is important to point out that citric acid, even if it is triprotic, has a pKa of 3.13, which means that it has a lower impact at lower concentrations; in addition, it is a known chelate, and it can form soluble conjugates with lignin, increasing its solubility at higher concentrations. It is worth mentioning that the yield obtained was high, but the purity of the lignin (83.98%) was lower than in the present study (90.35 ± 1.01%). For all the other acids, it is possible to suggest that the differences in yield obtained in the present study are associated with the amount of available total dry solids in the black liquor [43] and with the pH used in the precipitation. It was hypothesized that the amount of lignin precipitated with the lowest pH would be higher [32]; however, this was not verified. The available literature shows better performances in precipitation using a higher pH of between 3 and 5 depending on the organic acid chosen, which is also sometimes associated with an increase in temperature [26,28,43]. Furthermore, some studies did not disclose the characteristics of the black liquor, especially the total of dry solids, which may contribute to higher yields, making a direct comparison regarding its precipitation performance with different organic acids harder.

### 3.2. Lignin Characterization

#### 3.2.1. Chemical Characterization

**Figure 3:**
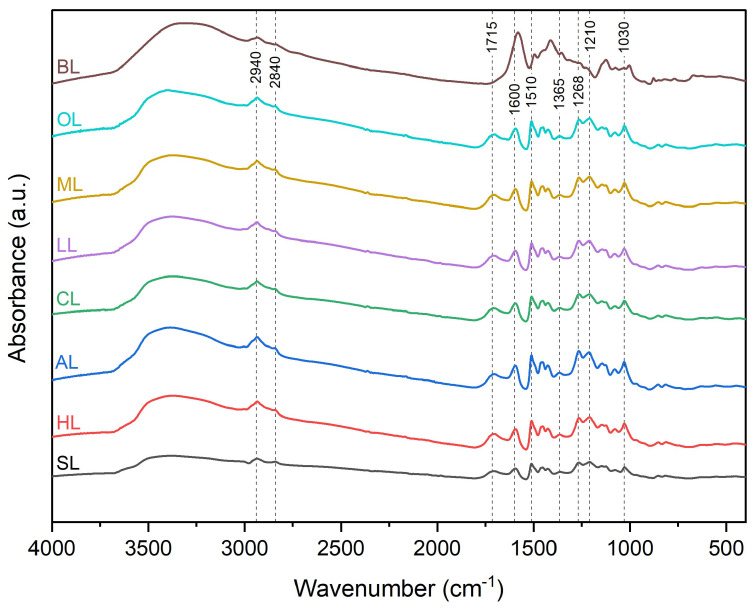
FTIR spectra of lignins and black liquor.

FTIR is a useful technique that contributes to identifying the complex structure of lignin. Figure 3 presents the spectra of the black liquor and the precipitated lignins, where it is possible to identify some of the main functional groups. The literature shows that bands between 3700 and 3000 cm^−1^ are associated with aliphatic, phenolic, and aromatic OH groups stretching vibration [44]. Different content of hydroxyl groups can explain the difference in intensity of these bands present on the lignin, but also by the moisture content. Although the conditioning of lignins is similar, moisture absorption can vary depending on the structure of the lignin. All samples present bands around 2940 and 2840 cm^−1^ related to the symmetrical and asymmetrical C-H stretching of the methyl and methylene groups, respectively. In the context of black liquor, the spectral feature at 2840 cm^−1^ appears as a broad band rather than a distinct band, and this characteristic is noticeable in lignins precipitated with inorganic acids, such as SL and HL. Da Silva and co-authors also observed this phenomenon in their research [26]. Another band at 1715 cm^−1^ is present in the lignin spectra but not in that of black liquor. This band is associated with a C=O stretch of non-conjugated carboxylic acids [26,42]. Bands from 1600 cm^−1^ to 1510 cm^−1^ are associated with C-H of aromatic ring stretching and deformational vibrations [42,45]. A noticeable shift toward lower wavelengths is observed in the case of these two bands in black liquor; furthermore, these bands seem more intense for the black liquor. Two other little bands are visible between 1460 and 1400 cm^−1^ and can be associated with C-H deformation in -CH_3_ and -CH_2_. The absorbance band at 1365 cm^−1^ can be interpreted as the C-H bond of the -CH_3_ groups of the syringyl unit. This band is slightly more pronounced in the case of lignins precipitated with organic acids. The band in the region of 1268 cm^−1^ is associated with C-O and C=O bonds, and the one located at 1210 cm^−1^ is identified as an aliphatic hydroxyl group [46]. Absorbance bands between 1150 and 1060 cm^−1^ can be associated with stretching C-O bonds. Finally, the band observed at 1030 cm^−1^ can be associated with C–H deformations of the syringyl cycle [44].

The results of the total phenolic content and the number of hydroxyl moles per gram of lignin are shown in Table 2. The difference between the precipitated lignins is not considerable. However, the ones precipitated with organic acids show a higher amount of total phenols and, consequently, a greater quantity of OH in their structure. This characteristic will enhance the reactivity of lignin. In their work, Da Silva and co-workers [26] deduced from their results that using a strong organic acid resulted in a lower content of total phenols. This assumption was not verified in our case, which is likely due to differences in black liquor source and secondary reactions occurring during precipitation. For instance, hardwood black liquor contains more β-O-4 linkages, which are more susceptible to cleavage during pulping, leading to more free phenolic groups [37]. However, stronger acids may promote condensation reactions during precipitation, which could explain the reduction in free phenols observed by Da Silva and co-workers [26]. In contrast, softwood black liquor is inherently more condensed than hardwood from the start [47], making it less prone to additional condensation during acid precipitation. This structural difference may explain why our results do not align with those of Da Silva and co-workers [26].

Each lignin’s antioxidant properties (IC50) were studied, and the values are reported in Table 2. Precipitated lignins have similar values, either precipitated with inorganic or organic acids, varying between 27.35 (AL) and 36.82 µg/mL (SL). The IC_50_ values measured for commercial antioxidants were 7.76 ± 0.12 and 5.78 ± 0.04 µg/mL for BHT and Trolox, respectively. As a reminder, a low IC_50_ value means that a small quantity of compound is sufficient to exhibit effective antioxidant activity. Consequently, precipitated lignins presented a lower antioxidant activity than commercial antioxidants. The IC_50_ is higher for lignin precipitated with sulfuric acid (36.82 ± 1.17 µg/mL). According to some studies [48,49], lignin should have a high quantity of phenolic hydroxyl and methoxy groups for effective antioxidant activity, low aliphatic hydroxyl groups, and low polydispersity and molecular weight values. In the present study, no such trend was observed. The results of IC_50_ found in the present work were not similar to those presented by Da Silva and co-workers [26] for the same acids, for instance. Differences in the black liquors could explain this and the reaction duration; Da Silva and co-workers 15 used 24 h, while in the present work, the precipitation took between 1 and 2 h, interrupted by neutralization after that period.

The GPC results are presented in Table 3. The number-average molecular weight (M_n_) varies between 2042 and 3148 g/mol, and the weight-average molecular weight (M_w_) varies between 14,024 and 17,358 g/mol. The values for these weights can be due to the process used in the industry that resulted in the black liquor. During the cooking stage of the Kraft process, the β-aryl bonds are cleaved, resulting in low molar weights [50]. Overall, the M_n_ for Kraft lignins is between 1000 and 3000 g/mol [21]. The values reported here are, therefore, within this range. The degree of acidity is another important factor to consider when assessing molecular weights. A strongly acidic environment facilitates the cleavage of more chemical bonds, reducing the molecular weight. This trend is apparent in some of the lignin samples analyzed in this study. For example, SL, obtained after precipitation with sulfuric acid (a strong acid), showed lower values for M_w_ and M_n_ than AL, which was precipitated with acetic acid (a weaker acid). It should also be noted that these values are considerably higher than those Da Silva and co-workers reported [26]. This variance may be explained by the difference in tree species between the two studies. Softwoods mainly comprise guaiacyl units, whereas hardwoods comprise guaiacyl (G) and syringyl (S) units [21]. The guaiacyl units can form non-cleavable C-C bonds during the Kraft process, which explains the higher molecular weights of softwoods [51,52]. Polydispersity (Đ) is high, from 5.51 to 6.87, indicating a wide range of molecular weights.

Quantitative acid hydrolysis was used to assess lignin purity. The results obtained are in Table 4. SL is the lignin with the lowest acid-insoluble lignin (AIL) content (77.66% for SL versus > 85% for the others). The results for carbohydrate content show lower values for SL and CL. However, these differences are not remarkable, and the carbohydrate content does not exceed 3.76% (AL). Thus, except for the case of acetic acid, lignins precipitated with organic acids showed comparable and even better purity than lignin obtained with sulfuric acid. Among the organic acids, OL has the highest purity (93.7% of lignin), and LL is the lowest (86.25%). According to a study by Yasuda and collaborators [53], acid-soluble lignin (ASL) comprises hydrophilic lignin derivatives and low-molecular-weight degradation products. As explained previously, using a strong acid leads to low-molecular-weight molecules. The lower quantities of ASL for LL, AL, and ML can be explained by the strength of the acid used, leading to higher molecular weight lignins.

The amount of ash, volatiles, and fixed carbon, which have a direct influence on lignin purity, was determined. Lower ash content contributes to higher lignin purity. The results are shown in Table 4. First, a significant difference in ash content was observed between SL (3.28%) and lignins precipitated with organic acids (less than 0.50%). This difference in ash content can be attributed to the potential presence of sulfur and the formation of salts during precipitation with sulfuric acid. In contrast, precipitation with organic acids yielded lignin samples with an ash content similar to that obtained by the LignoBoost^®^ process. A previous study [24] reported that this process produced high-purity lignins with ash contents ranging from 0.3% to 1.2%. In addition, the measured fixed carbon content exceeded 47%, indicating that the lignins obtained have favorable characteristics as precursors for carbon-based products [26].

Within the lignin structure, ^31^P NMR determined the amount of hydroxyl groups present. Depending on the type and quantity of hydroxyl groups, the reactivity and solubility of lignin are modified. All these values are listed in Table 5. The values obtained by NMR display similarities with those obtained using the Folin–Ciocalteu method. Indeed, lignins precipitated with citric or lactic acid have more hydroxyl groups than lignins precipitated with acetic or sulfuric acid. These results may indicate that the precipitation of the studied black liquor at pH 2 was not optimal for all organic acids, and acetic acid showed the worst balance between yield and acid consumption.

Regarding sulfuric acid, its strong acidity may have promoted some additional condensation, reducing the available hydroxyl group, as previously noted. Additionally, softwood is more condensed than hardwood from the start, presenting less available OH [47]. Nevertheless, these findings indicate that using certain organic acids at pH 2 for precipitation instead of sulfuric acid can yield lignins with a higher hydroxyl content and better reactivity.

Moreover, more guaiacyl OH groups can be observed than p-hydroxyphenyl and syringyl. These results are consistent with the literature and the fact that the analysis was carried out on maritime pine. Indeed, softwood lignin is mainly composed of G units with a low level of H units [21]. Syringyl units are generally not present in softwood lignin. However, other studies have also found low levels of syringyl hydroxyl groups during the ^31^P NMR analysis of softwood lignin [54].

#### 3.2.2. Thermal Characterization

**Figure 4:**
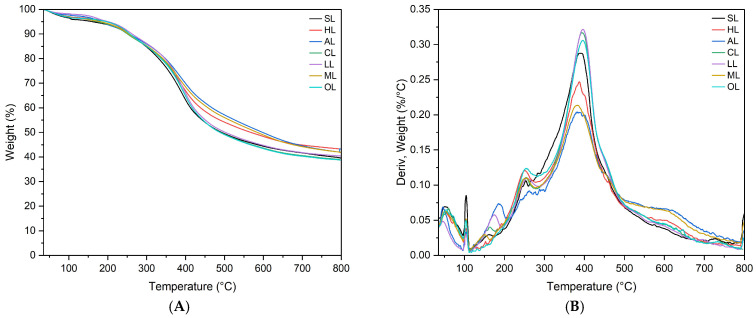
Thermogravimetric (TGA) (**A**) and derived thermogravimetric curves (DTG) (**B**) of lignins.

The thermal behavior of precipitated lignins was evaluated by thermogravimetric analysis under a nitrogen atmosphere. The results obtained before the gas change are shown in Figure 4. The first loss of mass corresponding to moisture evaporation is visible at 50 °C, with a major peak at 100 °C. A more important water loss can be noticed for sample SL. According to Da Silva and colleagues [26], the second phase of mass loss visible between 150 and 185 °C is probably due to fatty acids and the dehydration of the OH of the benzyl groups. The fragmentation of the β-aryl and α-aryl inter-unit bonds can be observable at 250 °C; this mass loss is almost invisible for sample AL. The maximum mass loss is at 395 °C and corresponds to the cleavage of the methyl-aryl ether bonds [26,55,56]. The order of mass loss at this stage from largest to smallest are LL, CL, OL, SL, HL, ML, and AL. The main mass loss occurs up to 500 °C, with a decrease in sample weight of around 48%. A temperature higher than 500 °C leads to decomposition and condensation reactions of the aromatic rings [52,56,57]. The final residual weight was between 38 and 43%.

## 4. Conclusions

This work describes the process of lignin precipitation from maritime pine black liquor at pH 2. To reduce dependence on the environmentally hazardous sulfuric acid (H_2_SO_4_) typically used in the precipitation phase, organic acids (acetic, citric, lactic, malic, and oxalic acids) were used as substitutes as sustainable alternatives. The precipitated lignins underwent detailed chemical and thermal characterization to assess their properties and structural features. The results showed that, in general, organic acids produced lignin with higher purity and higher yields compared to sulfuric acid. In addition, a notable correlation was observed between acid strength and molecular weight, indicating a reduction in molecular weight when using stronger acids such as H_2_SO_4_. Lignins precipitated with some organic acids showed increased hydroxyl groups, increasing their reactivity towards other compounds. However, the purity was considerably high and may have been acceptable as a compromise, considering valorization. Furthermore, a wider pH range should be investigated for optimal precipitation for the different acids studied.

These findings suggest that using organic acids for lignin precipitation appears to be a viable alternative for reducing the use of H_2_SO_4_. The choice of organic acid depends on the desired application; however, it can be noted that the use of oxalic acid seems to be a good compromise between the quantity of acid used, the yield, the number of hydroxyl groups, and the molar mass. This type of lignin can be used as a raw material in future products, and thanks to its fascinating properties (number of OH groups and molar mass), it can be chemically modified for greater reactivity.

## Figures and Tables

**Table 1:** Characteristics of black liquor used in the present study.

Parameter	Value	Unit
Density	1.14 ± 0.00	g/mL
pH	12.77 ± 0.46	-
Total dry solid	21.89 ± 0.06	(%)
Acid-soluble lignin	6.66 ± 0.04	(%)
Acid-insoluble lignin	27.31 ± 0.36	(%)
Carbohydrates	10.97 ± 0.13	(%)
Volatiles	27.13 ± 0.31	(%)
Fixed carbon	21.71 ± 1.47	(%)
Ash	47.16 ± 0.85	(%)

**Table 2:** Amount of lignin obtained, hydroxyl content, IC_50_ values, and total phenolic content of precipitated lignins (SL, HL, AL, CL, LL, ML, OL).

	g of Lignin Obtained/ 100 mL of Black Liquor	OH Content (mmol OH/g Dry Lignin)	IC_50_ (µg/mL)	Total Phenolic Content (µg GAE/g Dry Lignin)
SL	7.41 ± 0.09	0.67 ± 0.06	36.82 ± 1.17	285.01 ± 25.40
HL	8.07 ± 0.75	0.78 ± 0.03	28.72 ± 0.32	333.40 ± 12.33
AL	6.26 ± 0.56	0.70 ± 0.02	27.35 ± 0.24	298.38 ± 7.74
CL	7.45 ± 0.32	0.79 ± 0.00	29.95 ± 0.34	336.34 ± 1.30
LL	7.01 ± 0.49	0.82 ± 0.02	31.93 ± 0.35	348.39 ± 8.77
ML	7.62 ± 0.39	0.78 ± 0.02	32.65 ± 0.09	331.84 ± 10.17
OL	7.62 ± 0.03	0.73 ± 0.08	32.50 ± 0.91	331.84 ± 32.51

**Table 3:** Molecular weight distribution (M_n_, M_w_) and polydispersity (Đ) of precipitated lignins (SL, HL, AL, CL, LL, ML, OL).

	Mn (g/mol)	Mw (g/mol)	Đ
SL	2042	14,024	6.87
HL	2781	15,415	5.54
AL	3012	17,130	5.69
CL	2667	16,025	6.01
LL	2875	16,160	5.62
ML	3148	17,358	5.51
OL	2770	15,620	5.64

Mn: Number-average molecular weight. Mw: Weight-average molecular weight. Đ: Dispersity (M_w_/M_n_).

**Table 4:** Acid-insoluble lignin (AIL), Acid-soluble lignin (ASL), and composition in terms of carbohydrates, volatiles, fixed carbon, and ash for precipitated lignins (SL, HL, AL, CL, LL, ML, OL).

	AIL (%)	ASL (%)	Carbohydrates (%)	Volatiles (%)	Fixed Carbon (%)	Ash (%)
SL	77.66 ± 7.63	1.68 ± 0.06	3.25 ± 0.13	44.83 ± 1.20	51.53 ± 1.35	3.28 ± 0.07
HL	90.87 ± 0.61	1.63 ± 0.04	3.58 ± 0.02	45.56 ± 1.27	52.71 ± 1.51	0.31 ± 0.04
AL	89.76 ± 0.40	0.65 ± 0.09	3.76 ± 0.11	45.13 ± 0.27	54.08 ± 0.64	0.17 ± 0.02
CL	90.35 ± 1.01	1.77 ± 0.33	3.04 ± 0.02	48.16 ± 0.73	51.07 ± 0.38	0.32 ± 0.02
LL	85.20 ± 5.24	1.05 ± 0.13	3.37 ± 0.25	40.49 ± 2.86	54.95 ± 2.3	0.10 ± 0.02
ML	92.00 ± 0.99	1.11 ± 0.17	3.53 ± 0.13	52.14 ± 2.55	52.14 ± 2.55	0.49 ± 0.02
OL	92.43 ± 0.20	1.27 ± 0.05	3.38 ± 0.09	46.02 ± 0.91	47.60 ± 0.87	0.45 ± 0.03

**Table 5:** Hydroxyl types and S/G ratio obtained from ^31^P NMR.

		SL	HL	AL	CL	LL	ML	OL
OH types (mmol/g)	Aliphatic	1.57	1.97	1.41	2.70	1.91	1.59	1.86
	C_5_-substituted	2.57	2.93	1.75	3.35	2.84	2.71	2.6
	Guaiacyl	3.21	3.70	1.69	3.63	3.33	3.27	3.24
	*p-*hydroxyphenyl	0.03	0.08	0.09	0.11	0.07	0.06	0.02
	Syringyl	0.03	0.04	0.03	0.03	0.02	0.04	0.05
	Phenolic	5.81	6.70	3.53	7.08	6.24	5.81	5.85
	Carboxylic acid	0.27	0.31	0.15	0.28	0.19	0.27	0.20
S/G ratio		0.009	0.011	0.018	0.008	0.006	0.012	0.015

## Data Availability

The original contributions presented in this study are included in the article. Further inquiries can be directed to the corresponding author(s).
